# Phylogenetic analysis and molecular evolution of the dormancy associated MADS-box genes from peach

**DOI:** 10.1186/1471-2229-9-81

**Published:** 2009-06-27

**Authors:** Sergio Jiménez, Amy L Lawton-Rauh, Gregory L Reighard, Albert G Abbott, Douglas G Bielenberg

**Affiliations:** 1 Department of Horticulture, Clemson University, Clemson, SC 29634-0319, USA; 2 Department of Genetics & Biochemistry, Clemson University, Clemson, SC 29634-0318, USA; 3 Department of Biological Sciences, Clemson University, Clemson, SC 29634-0314, USA

## Abstract

**Background:**

Dormancy associated MADS-box (DAM) genes are candidates for the regulation of growth cessation and terminal bud formation in peach. These genes are not expressed in the peach mutant *evergrowing*, which fails to cease growth and enter dormancy under dormancy-inducing conditions. We analyzed the phylogenetic relationships among and the rates and patterns of molecular evolution within DAM genes in the phylogenetic context of the MADS-box gene family.

**Results:**

The peach DAM genes grouped with the *SVP*/StMADS11 lineage of type II MIKC^C ^MADS-box genes. Phylogenetic analyses suggest that the peach *SVP*/StMADS11-like gene family, which contains significantly more members than annual model plants, expanded through serial tandem gene duplication. We found evidence of strong purifying selection acting to constrain functional divergence among the peach DAM genes and only a single codon, located in the C-terminal region, under significant positive selection.

**Conclusion:**

Because all DAM genes are expressed in peach and are subjected to strong purifying selection we suggest that the duplicated genes have been maintained by subfunctionalization and/or neofunctionalization. In addition, this pattern of selection suggests that the DAM genes are important for peach growth and development.

## Background

MADS-box genes are a family of transcription factors found in animals, fungi, and plants and all contain a conserved DNA-binding domain [1]. MADS-box transcription factors play fundamental roles in plant development, as floral organ and meristems identity determination and transition from vegetative to reproductive growth regulation [2]. Animal, fungal and plant MADS proteins are classified into two main groups: type I and type II [1]. In plants, the first has been subdivided into M_α_, M_β _and M_γ _types based on the phylogenetic relationships among MADS-box domains [3].

Type II MADS-box proteins bind to DNA as dimers or higher order complexes and are also referred to as the MIKC-type due to their common structure of four domains: M domain, I region, K domain and C region. M represents the MADS domain involved in the DNA binding, is approximately 60 amino acids long, and contains an α-helix followed by a β-strand. The K domain is a coiled-coil structure that participates in protein-protein interaction [4], and is subdivided into three α-helix structures, K_1_, K_2_, and K_3_. The variable I region, consisting of about 30 amino acids, links the M and K domains. And finally, the C-terminal region continues the helix structure of the K_3 _subdomain and is the most variable region among family members. The C region functions in transcriptional activation of other factors and the formation of multimeric MADS-box protein complexes [4,5]. The MIKC-type genes can be further subdivided in two types based on intron-exon structure [6]: the MIKC^C ^and the MIKC*, also named M_δ _in Parenicova *et al*. [3].

Extensive gene duplication and subsequent modification in various MADS-box family lineages has resulted in diversified protein functions [7]. MADS-box transcription factors, besides being involved in floral organ specification, are also involved in several pathways of plant growth and development, such as fruit ripening, embryonic development, and vegetative development of root and leaves [6,8-11]. Studies of the evolution of MADS-box genes that act in non-floral aspects of plant development could yield general insights into the mechanisms behind functional diversification of developmental gene families [12].

One approach to examining the evolution of these gene families is to test for molecular signatures of natural selection. The ratio of nonsynonymous (dN) to synonymous (dS) substitution rates (dN/dS or ω) provides a sensitive test of natural selection. A statistically significant dN/dS ratio lower than, equal to, or greater than 1.0 can indicate purifying selection, neutral evolution and positive selection, respectively. Analysis of MIKC-type genes in *Arabidopsis *demonstrated periods of both positive selection and purifying selection [13]. Changes in coding sequences represented by these periods of selection, in both DNA-binding and non-DNA-binding regions of MADS transcriptions factors, seem to play important roles during phenotypic evolution of plants.

Besides PpAG1 [14], a *FUL*-like and a *SHP*-like [15], an *AP1*-like and a *PI*-like [16], and three *SEP*-like genes [17], six other MIKC-type genes have been described in peach [*Prunus persica *(L.) Batsch]. These genes, named dormancy associated MADS-box (DAM), are candidates for the regulation of growth cessation and terminal bud formation in peach [18]. The DAM genes are not expressed in the peach dormancy-incapable mutant *evergrowing *[18]. To study the divergence patterns and processes of these genes, identify the most closely related *Populus *sp. genes for homology studies, and test for redundancy resulting from recent shared duplication history, we performed phylogenetic and evolutionary analyses of the DAM genes as members of the MIKC-type lineage of the MADS-box gene family. We found that the PpDAM genes are *SVP*/StMADS11-like (*SHORT VEGETATIVE PHASE*), and were derived by tandem duplications. In addition, we identified significant patterns of sequence constraint in the PpDAM genes, suggesting a history of natural selection that removes amino acid-changing mutations in these genes.

## Results

### Phylogeny of *Arabidopsis*, peach and poplar MIKC^C^-type MADS-box gene

**Figure 1:**
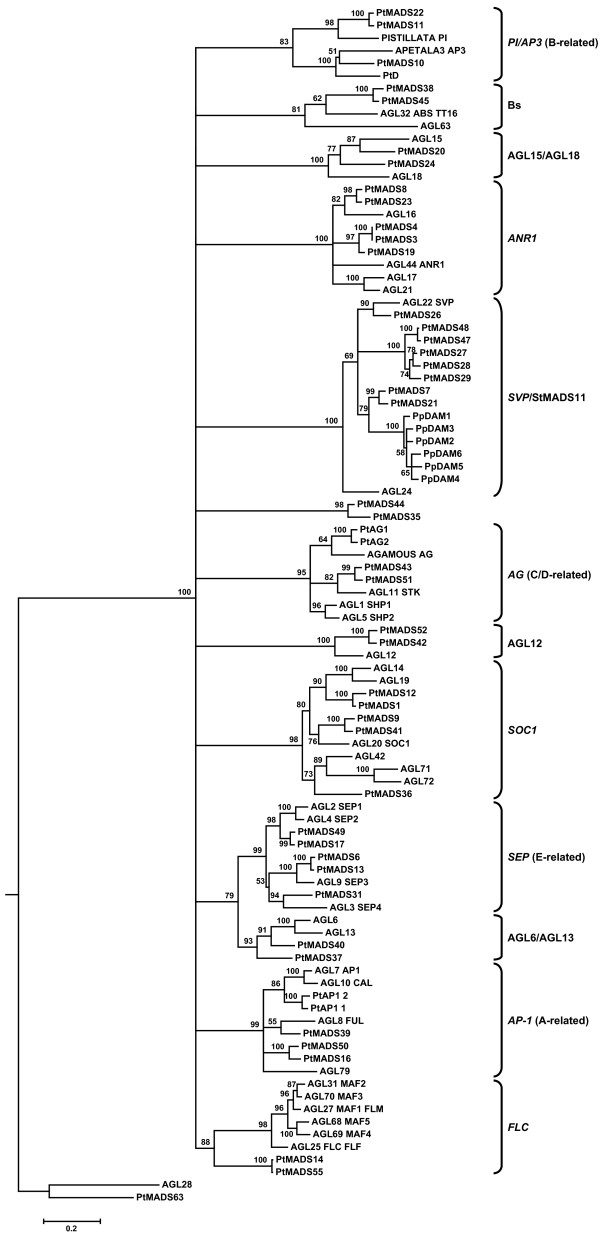
**Maximum likelihood rooted tree of 39 *Arabidopsis*, 6 peach and 48 poplar MIKC^C^-type MADS-box genes**. The tree was constructed using nucleotide sequences and a GTR+I+G evolution model. The AGL28 and PtMADS63 sequences were used as outgroups. The numbers at each interior branch indicate bootstrap support of 1000 replicates. Branches with less than 50% bootstrap support are collapsed. Branch lengths are proportional to the number of nucleotide changes.

We compared the *Arabidopsis *[3] and poplar [19] MIKC^C^-type MADS-box genes with the peach DAM genes to determine the phylogenetic relationship between these genes. A maximum likelihood tree was estimated using the M, I and K domains (Figure 1). Twelve major lineages could be resolved and were named according to the *Arabidopsis *gene conventions and the ABC-model classification [5,9,20]. All twelve lineages *PI/AP3 *(B-related), Bs, AGL15/AGL18, *ANR1*, *SVP*/StMADS11, *AG *(C/D-related), AGL12, *SOC1*, *SEP *(E-related), AGL6/AGL13, *AP1 *(A-related) and *FLC *clades, were defined with bootstrap values of at least 79 or higher.

The six PpDAM genes could be unambiguously classified within the *SVP*/StMADS11 group, together with 2 *Arabidopsis *(AGL22/*SVP *and AGL24) and 8 poplar (PtMADS7, PtMADS21, PtMADS26, PtMADS27, PtMADS28, PtMADS29, PtMADS47 and PtMADS48) MADS-box genes (Figure 1). The peach homologs formed a monophyletic group in this clade that was most closely related to the two poplar homologs, PtMADS7 and PtMADS21. However, the other poplar homologs grouped into sister clades or as orthologs to the *Arabidopsis *gene AGL22/*SVP*. Therefore, the estimated tree suggests a single common ancestor for all six peach DAM genes.

A Bayesian tree estimated using the same data showed a similar topology with higher resolution due to high support values in most of the nodes (Additional file 1), and a maximum parsimony tree showed similar topology and support (Additional file 2). Thus, in three different analyses, the peach DAM genes formed a monophyletic group within the *SVP*/StMADS11 clade.

### PpDAM sequence characterization

**Figure 2:**
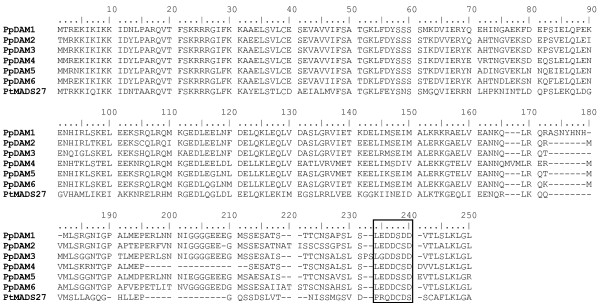
**Alignment of predicted amino acid sequences of the MIKC^C^-type MADS-box genes of peach**. Sequence comparison was obtained using Clustal X and utilized for tree estimation and ancestral reconstruction. Sequences included M, I, K and C domains. A poplar gene (PtMADS27) was included as outgroup.

**Figure 3:**
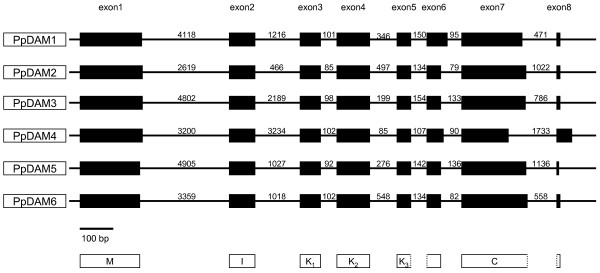
**Schematic overview of introns and exons of the six peach DAM genes**. Boxes represent exons scaled to number of base pairs. Lines represent introns. Introns are not drawn to scale, but size is indicated by number of base pairs.

The alignment of the complete PpDAM protein sequence revealed high amino acid sequence conservation among the peach MADS genes (Figure 2). The intron-exon structure is also conserved in all domains (Figure 3), with the exception of the C domain of PpDAM4, which contains a deletion of similar size to that present in PtMADS27. The modular domain organization of MIKC proteins is reflected in a conserved intron-exon structure [4].

By contrast with the exons, intron sequence similarity was low. Sequence length in most of the introns among the PpDAM genes was highly variable (Figure 3). This variability precluded meaningful intron sequence alignments, so we could not use the intron sequence to establish relationships among DAM genes.

### Phylogeny of PpDAM genes

**Figure 4:**
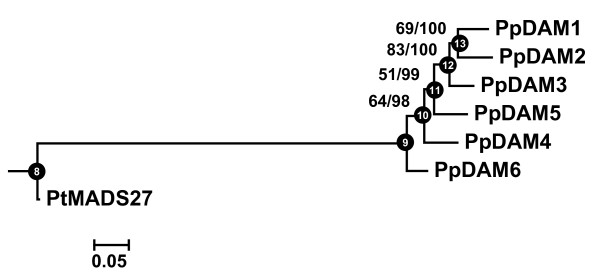
**Maximum likelihood rooted tree of 6 peach MIKC^C^-type MADS-box genes**. Tree was constructed using nucleotide sequences and an HKY evolution model. The PtMADS27 sequence was used as outgroup. The right numbers at each interior branch indicate maximum likelihood bootstrap support, and the left numbers indicate Bayesian clade credibility values of 1000 replicates. Branch lengths are proportional to the number of nucleotide changes. Black circles in the internal nodes correspond to ancestral sequences in the reconstruction experiment described in Figure 5.

To further resolve the phylogenetic relationships of the PpDAM genes, a maximum likelihood and Bayesian tree was estimated using the aligned M, I, K and C domains (Figure 4). The variable C domain was added into the analysis to increase the number of informative characters and therefore improve the resolution of the PpDAM tree. The best fit estimated phylogenetic tree suggested that the PpDAM genes were derived from serial duplication events in the following order: PpDAM6, PpDAM4, PpDAM5, PpDAM3, PpDAM2, and finally PpDAM1. Separate maximum parsimony tree (Additional file 3) generated the same topology as obtained by maximum likelihood and Bayesian trees. However, confidence levels differed: Bayesian posterior probabilities showed high support in all nodes, whereas bootstrap values of maximum likelihood and parsimony trees were low between PpDAM6 and PpDAM4, and also between PpDAM4 and PpDAM5.

### Evolutionary analysis of *Arabidopsis*, peach and poplar MIKC^C^-type MADS-box genes

Models of the molecular evolution of the entire MIKC^C^-type group from *Arabidopsis*, poplar, and peach were statistically tested using the same alignment and the inferred best fit phylogenetic tree estimated using maximum likelihood (Figure 1).

To test for statistically different rates of sequence evolution among branches or sites, data were analyzed using algorithms implemented in PAML 4. A codon substitution free-ratio model (model = 1, M) that allows different dN/dS rate ratios among branches fit the data better than the model that assumed a homogeneous mean dN/dS rate ratio for all lineages (model = 0, M0; Table 1). To examine how dN/dS rate ratios differed among codon positions, we compared models M0 and M3, which allows 3 different rates among sites, and found it to produce a significantly better fit (*P *< 0.001). Purifying selection (ω_0 _= 0.02, ω_1 _= 0.12, ω_2 _= 0.36; Table 1) was found. However, no positive selection was found at any site. To test if positive selection promoted divergence between genes, the codon substitution models that allow positive selection (M2a and M8) and that assumed nearly neutral selection (M1a and M7) were compared (M2a vs. M1a and M8 vs. M7; Table 1). In both cases, we found no significant evidence of positive selection.

**Table 1:** Evolutionary analysis of the MIKC^C^-type MADS-box *Arabidopsis*, poplar and peach genes

Site model: One ω ratio for each branch						
Nested Model pairs	dN/dS^a^	Parameters estimates^b^	PSS^c,d^	ℓ	Model comparison [Δdf]	2ℓ^d^

M: free ratio	several	-	-	-17495.098	Model1 vs. M0 [169]	590.92***

Site model: One ω ratio for all branches						

Nested Model pairs	dN/dS^a^	Parameters estimates^b^	PSS^c,d^	ℓ	Model comparison [Δdf]	2ℓ^d^

M0: one ratio	0.137	ω = 0.14		-17790.560		
M3: discrete (k = 3)	0.155	p_0 _= 0.28, p_1 _= 0.46, p_2 _= 0.26 ω_0 _= 0.02, ω_1 _= 0.12, ω_2 _= 0.36	None	-17165.244		
M3: discrete (k = 2)	0.151	p_0 _= 0.47, p_1 _= 0.53 ω_0 _= 0.04, ω_1 _= 0.25	None	-17319.365	M3 vs. M0 [4]	1250.63***
M1a: nearlyneutral	0.341	p_0 _= 0.76, p_1 _= 0.24 ω_0 _= 0.13, ω_1 _= 1.00	Not allowed	-17592.704		
M2a: positive selection	0.341	p_0 _= 0.76, p_1 _= 0.05, p_2 _= 0.19 ω_0 _= 0.13, ω_1 _= 1.00, ω_2 _= 1.00	None	-17592.704	M2a vs. M1a [2]	0
M7: beta	0.166	p = 0.61, q = 2.76	Not allowed	-17168.752		
M8: beta & ω>1	0.166	p_0 _= 1.00, p_1 _= 0.00 p = 0.61, q = 2.76, ω = 1.00	None	-17168.752	M8 vs. M7 [2]	0

						
Branch-specific model: ancestral *SVP*/StMADS11 clade						

Nested Model pairs	dN/dS^a^	Parameters estimates^b^	PSS^c,d^	ℓ	Model comparison [Δdf]	2ℓ^d^

Model A1	-	p_0 _= 0.68, p_1 _= 0.22, p_2_+ p_3 _= 0.10 ω_0 _= 0.13, ω_1 _= 1.00, ω_2 _= 27.63	16**, 26, 124*, 125**, 139, 151, 169	-17582.792	A1 vs M1a [2]	19.82***
Model A2	-	p_0 _= 0.59, p_1 _= 0.19, p_2_+ p_3 _= 0.17 ω_0 _= 0.13, ω_1 _= 1.00, ω_2 _= 1.00	N/A	-17586.128	A1 vs A2 [1]	6.67**
Model B	-	p_0 _= 0.41, p_1 _= 0.45, p_2_+ p_3 _= 0.14 ω_0 _= 0.04, ω_1 _= 0.25, ω_2 _= 9.54	4, 7, 16*, 26, 50, 124**, 125*, 127, 139, 169, 172, 173	-17312.590	B vs M3(k = 2) [2]	13.55**

						
Branch-specific model: ancestral DAM clade						

Nested Model pairs	dN/dS^a^	Parameters estimates^b^	PSS^c,d^	ℓ	Model comparison [Δdf]	2ℓ^d^

Model A1	-	p_0 _= 0.67, p_1 _= 0.22, p_2_+ p_3 _= 0.11 ω_0 _= 0.13, ω_1 _= 1.00, ω_2 _= 1.00	N/A	-17592.339	A1 vs M1a [2]	0.73
Model A2	-	p_0 _= 0.67, p_1 _= 0.22, p_2_+ p_3 _= 0.11 ω_0 _= 0.13, ω_1 _= 1.00, ω_2 _= 1.00	N/A	-17592.339	A1 vs A2 [1]	0
Model B	-	p_0 _= 0.39, p_1 _= 0.44, p_2_+ p_3 _= 0.17 ω_0 _= 0.04, ω_1 _= 0.25, ω_2 _= 0.40	N/A	-17318.615	B vs M3(k = 2) [2]	1.5

^a^Average over all sites.

^b^p_i_: proportion of sites. p, q: parameters of the β distribution.

^c^PSS: Number of positively selected sites. Naïve empirical Bayes was used in M3 and Bayes empirical Bayes in M2a and M8.

^d^Asterisks indicate significance: * *P *< 0.05, ** *P *< 0.01, *** *P *< 0.001.

Because positive selection often occurs only during short stretches of evolutionary history, detection of statistically significant patterns consistent with past positive selection events can be difficult when considering average measures of selection among lineages. The free-ratio model (model = 1, M) indicated that selection was not homogeneous among branches. Several branches of the *SVP*/StMADS11 clade showed a considerable number of changes that could be related to differential selection pressure. Thus, variation in selection pressure was evaluated for the branches that lead to *SVP*/StMADS11 and DAM clade genes by comparing models that allow positive selection and different rates among sites with nearly neutral models (Table 1). Significant positive selection (ω_2 _= 27.63 for 10% of sites) was found in the basal branch of *SVP*/StMADS11 clade (*P *< 0.001 for A1 vs. M1a comparison). Three significant positive selected sites were also found in this ancestor: one in the M and two in the K domain. However, no significant detectable positive selection was observed in the basal branch of DAM clade (Table 1).

### Evolutionary analysis of PpDAM group

Models of molecular evolution within the PpDAM genes were tested for best fit using the alignment and maximum likelihood tree (Figure 4). Similar results were obtained by two programs: MEGA 4 and PAML4. Estimation of pairwise dN and dS rates using MEGA 4 showed significant purifying selection and no significant positive selection for each of the six sequences (data not shown).

A codon substitution free-ratio model (model = 1, PAML 4) that allows different dN/dS rate ratios among branches did not fit the data better than the model that assumed a mean dN/dS rate ratio for all the lineages (model = 0, M0; Table 2). To evaluate whether there was dN/dS rate ratio variation among codon positions, models M0 and M3 were compared. As with the entire MIKC^C^-type tree, the model M3, which allows three different rates among sites, was a significantly better fitting model (*P *< 0.001) within the PpDAM clade. Approximately one-half of the sites had patterns consistent with purifying selection, and half were more consistent with neutral sequence evolution (ω = 0.90; Table 2). Three percent of sites showed patterns with statistically significant positive selection (ω = 4.66). To test if a positive selection model could explain the divergence between PpDAM genes, the codon substitution models that allow positive selection (M2a and M8) and that assumed nearly neutral selection (M1a and M7) were compared (Table 2). In the first case (M2a vs. M1a), no amino acid showed significant evidence of positive selection. However, when a β distribution of ratios was applied (M8 vs. M7), the pattern suggesting positive selection was statistically significant (*P *< 0.05) although the percentage of sites contributing to this significant deviation from neutrality was very low (4%). The overall analysis of PpDAM genes showed that most sites were either highly conserved with a dN/dS rate ratio close to 0 or nearly neutral.

**Table 2:** Evolutionary analysis of the MIKC^C^-type MADS-box genes of peach

Site model: One ω ratio for each branch						
Nested Model pairs	dN/dS^a^	Parameters estimates^b^	PSS^c,d^	ℓ	Model comparison [Δdf]	2ℓ^d^

M: free ratio	several	-	-	-2519.205	Model1 vs. M0 [11]	11.63

Site model: One ω ratio for all branches						

Nested Model pairs	dN/dS^a^	Parameters estimates^b^	PSS^c,d^	ℓ	Model comparison [Δdf]	2ℓ^d^

M0: one ratio	0.434	ω = 0.434		-2525.019		
M3: discrete (k = 3)	0.611	p_0 _= 0.51, p_1 _= 0.46, p_2 _= 0.03 ω_0 _= 0.10, ω_1 _= 0.90, ω_2 _= 4.66	62, 79, 185, 218, 228, 239*	-2486.684	M3 vs. M0 [4]	76.67***
M1a: nearlyneutral	0.509	p_0 _= 0.55, p_1 _= 0.45 ω_0 _= 0.11, ω_1 _= 1.00	Not allowed	-2489.546		
M2a: positive selection	0.632	p_0 _= 0.54, p_1 _= 0.43, p_2 _= 0.03 ω_0 _= 0.12, ω_1 _= 1.00, ω_2 _= 5.15	62, 79, 185, 218, 228, 239	-2486.782	M2a vs. M1a [2]	5.53
M7: beta	0.476	p = 0.36, q = 0.40	Not allowed	-2491.716		
M8: beta & ω > 1	0.593	p_0 _= 0.96, p_1 _= 0.04 p = 0.44, q = 0.55, ω = 3.94	62, 79, 81, 83, 185, 218, 228, 239*	-2487.434	M8 vs. M7 [2]	8.56*

^a^Average over all sites.

^b^p_i_: proportion of sites. p, q: parameters of the β distribution.

^c^PSS: positively selected sites. Naïve empirical Bayes was used in M3 and Bayes empirical Bayes in M2a and M8.

^d^Asterisks indicate significance: * *P *< 0.05, ** *P *< 0.01, *** *P *< 0.001.

### Ancestral reconstruction of a positively selected region in PpDAM

**Figure 5:**
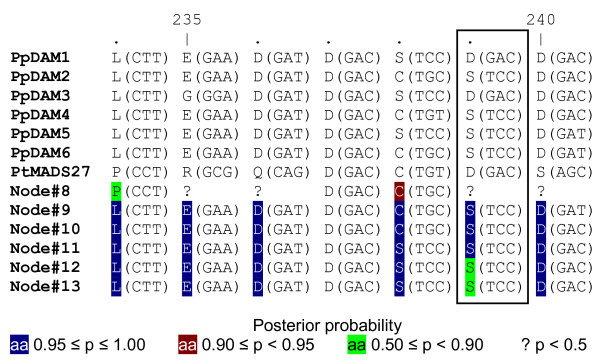
**Ancestral reconstruction in the region of the positively selected site 239 in PpDAM genes**. Sequence corresponds with the rectangular region of Figure 2. Ancestral names of sequences correspond to internal nodes of Figure 4. The posterior probability for the reconstructed amino acid at an internal node is indicated. Non-variable sites are not highlighted.

The Naïve empirical Bayes of M3 and M8 models suggested one potential site under significant positive selection (Table 2): a serine-aspartate change of the C-terminal region (amino acid position 239 in Figure 2). A maximum likelihood-based estimated reconstruction of ancestral sequences around this site is shown in Figure 5. Identical changes (S to D) at amino acid 239 are present in PpDAM1 and PpDAM3. In this section of the alignment, ancestral sequences of PpDAM genes shared the same nucleotides with a probability higher than 0.95, except near PpDAM1 and PpDAM3 genes at the potential positively selected site. However, ancestral sequences of PpDAM genes did not shared the same nucleotides (probability higher than 0.90) in the non-positive selected positions 238 and 240 around this site (Figure 5).

## Discussion

Phylogenetic analyses were used to test alternative models of sequence evolution in the peach DAM genes, members of the MADS-box family. Expression of the DAM genes is lost in the *evergrowing *peach mutant, which does not form terminal vegetative buds in response to dormancy inducing conditions. Disruption of gene function for one or more of these genes may be responsible for the non-dormant phenotype of the mutant [18].

Phylogenetic relationships among MIKC^C^-type genes of *Arabidopsis *and poplar were similar to those obtained previously in other studies [3,13,19]. MIKC^C^-type genes were resolved in 12 subfamilies although the order of divergence among them was unclear. Nevertheless, the peach DAM genes were unambiguously recognized as *SVP*/StMADS11-like genes.

The *SVP*/StMADS11 group appears to have expanded in the few perennials for which we have comprehensive data: poplar (eight) and grapevine (five) carry an increased number of *SVP*/StMADS11 genes relative to *Arabidopsis *(two) and other annual models such as tomato (two) and rice (three) [19,21]. The six DAM genes found in the peach follow the trend of increased *SVP*/StMADS11 genes in perennial species. This expansion in three phylogeneticaly distinct species could indicate that perennials use *SVP*/StMADS11 genes for functions that are no longer required or needed in annual models. These traits could include the formation of floral and vegetative bud structures, regulation of endodormancy cycling, or regulation of the juvenile to mature transition. The *SVP*/StMADS11 genes, together with other expanded genes from *ANR1*, *SOC1 *and AGL32 families, are interesting targets for investigating the functional requirements for the evolution of perenniality [19].

Several *SVP*/StMADS11-like genes have been associated with the vegetative to reproductive meristem transition. In *Arabidopsis*, the AGL22/*SVP *gene acts as flowering repressor [22], whereas its close homolog AGL24 has an opposite function [23]. In rice, OsMADS22, OsMADS47 and OsMADS55 act as negative regulators of brassinosteroid responses and modulators of meristem identity [24,25]. However, in tomato, the gene *JOINTLESS *is involved in leaf and abscission zone development, in addition to the flowering time regulation [26,27].

In perennial species where *SVP*/StMADS11 genes have been found, the genes are expressed in most vegetative tissues and often in bud tissues. In potato, StMADS11 and StMADS16 are preferentially expressed in vegetative tissues [28]. Similarly, IbMADS3 and IbMADS4 genes of sweet potato are preferentially expressed in root tissue [29]. In *Paulownia kawakamii*, PkMADS1 can act as regulator of shoot morphogenesis [30]. In poplar, at least one of the *SVP*/StMADS11-like genes is expressed in cambium tissue [19]. The five grapevine *SVP*/StMADS11-like genes are preferentially expressed in bud tissue, although two of them are also detected in vegetative organs [21]. A raspberry gene putatively encoding an *SVP*/StMADS11-like transcription factor [31], a Japanese apricot *SVP*/StMADS11-like [32] and the peach DAM genes [18] are all expressed in bud tissues. The vegetative and/or bud localization of expression of these genes would support the hypothesis that a perennial habit of growth would require an increased sophistication of the regulatory pathways devoted to controlling dormancy and growth cycles of dormant buds, influencing architecture and survival of unfavourable growing conditions.

Our phylogenetic analyses suggest that the peach DAM genes were derived from serial duplications of a common ancestor. Duplicated genes can be produced either by genome, segmental or tandem gene duplication. Gene duplications are especially prevalent in plants [7], and so the relative proportions for each of these fates is of significant interest. In poplar, the presence of several gene regions with fewer than three genes in each gene cluster [19] suggest that the expansion of the *SVP*/StMADS11-like genes was a consequence of combined whole-genome [33] or segmental duplication with tandem gene duplications [19]. By contrast, the six peach DAM genes are clustered in one locus of the linkage group 1 (LG1) of the general *Prunus *genetic map [18] and form a monophyletic clade in our analyses. Monophyly strongly suggests that expansion of the peach DAM gene family occurred by repeated rounds of tandem duplication. Thus, the duplications in both poplar and peach provide examples of two different pathways leading to the origin and maintenance of an elevated number of duplicated genes. The plant groups in which these expansions have occurred suggests that *SVP*/StMADS11-like genes could play roles in perennial state or bud development.

Duplicated genes can have several alternative long-term fates: nonfunctionalization, subfunctionalization or the much rarer neofunctionalization [34-37]. Overall, the MIKC^C^-type genes sampled in this study have substitution patterns consistent with purifying selection. However, several branches of the MIKC^C^-type gene tree, including the branch leading to the *SVP*/StMADS11-like genes, appear to be under strong positive selection. We found three potential positively-selected sites that could have modified the DNA binding and protein interaction properties of the *SVP*/StMADS11-like genes. By contrast, the evolution of the PpDAM genes has involved strong purifying selection, suggesting important functions in peach trees that rely upon the retention of specific sequences. The *Arabidopsis *type II MADS-box genes may have been affected by sporadic positive selection at the origin of new functions followed by strong functional constraint [13,38], and our observations in peach and poplar are consistent with a similar model for evolution in the *SVP*/StMADS11 clade. Indeed, since the dN/dS rate ratio among branches of the PpDAM clade tree was not significantly different, the selection intensity and direction appears to have been constant in all PpDAM genes. The patterns of strong purifying selection observed in all six PpDAM genes could also suggest a lack of functional redundancy among the genes, despite similarity in coding sequence. Four different seasonally dependent expression patterns among the PpDAM genes [39] are consistent with independent roles for these gene groups in growth and development. Therefore subfunctionalization and/or neofunctionalization likely contribute to the maintenance of this paralogous set of genes, although overlapping redundancy may also occur.

Purifying selection appears to be strongest in the MIK region. The relaxation of functional constraint in the C-terminal region is consistent with previous studies that suggest the C-terminus tends to be more divergent than the MIK region [3,36,40]. Within the C-terminal region of the DAM genes we identified a potential positively-selected site. This position had a posterior possibility higher than 0.95 and could be related to the suspected functions of the C-terminal region: stabilization of the interactions mediated by the K domain, formation of DNA-binding homodimers, activation of transcription, or contribution to multimer or higher-order complex formation [4,5,41]. Functional studies of motifs and particular amino acids have been performed in the C-terminal domain of B-family proteins [42,43], but similar information is not available for the *SVP*/StMADS11 family.

## Conclusion

Our results demonstrate that the peach DAM genes diverged sequentially by tandem duplications from a common ancestor related to the two poplar genes, PtMADS7 and PtMADS21, which are more closely related to the peach DAM genes than are other *SVP*/StMADS11 family genes. In addition, the nucleotide substitution patterns and rates of evolution in DAM homologs suggest strong functional constraints.

## Methods

### Sequence collection

*Arabidopsis *MADS-box genes of MIKC^C^-type were parsed from the *Arabidopsis *nucleotide dataset generated by The Arabidopsis Information Resource (TAIR,[44]) using gene identification numbers reported by Parenicova *et al*. [3]. Peach genes DAM1 to 6 [GenBank: DQ863253, DQ863255, DQ863256, DQ863250, DQ863251 and DQ863252, respectively] were cloned by our laboratory [18]. Poplar MADS-box genes of the same type were parsed from *Populus trichocarpa *genome dataset v1.1 [45] using gene identification numbers reported by Leseberg *et al*. [19] (see supplementary data of Leseberg et al [19] for correspondence with other nomenclatures, such as the one proposed by De Bodt [46]).

### Phylogenetic analyses

An initial phylogenetic tree was estimated for MADS-box genes from *Arabidopsis*, peach and poplar. The Mα-type MADS-box genes AGL28 from *Arabidopsis *and PtMADS63 from poplar were used as outgroups to root the MIKC^C^-type MADS-box gene phylogeny [3,19]. Nucleotide sequences from the M, I and K domains were aligned with reference to the corresponding amino acid alignment using Clustal X [47] and appropriate settings (pairwise alignment parameters for gap opening 22.5 and for gap extend 0.45, multiple alignment parameters for gap opening 12.5 and for gap extend 0.25). This alignment was then manually refined and end trimmed using BioEdit version 7.0.5.3 [48]. Poplar genes without identifiable I and K domains were excluded from these analyses. The resulting alignment is presented in the Additional file 4. Maximum likelihood analysis using the nucleotide matrix was conducted using PAUP* 4.0b10 [49]. Trees were estimated using the Tree Bisection-Reconnection (TBR) branch swapping algorithm, and the GTR+I+G evolution model parameters and the best fit tree was assessed using heuristic searches. The GTR+I+G substitution model was the best fit model as tested using Modeltest 3.7 [50] hierarchical likelihood ratio test (hLRTs), corrected Akaike information criterion (AICc) and Bayesian information criterion (BIC). Bootstrap resampling [51] was performed in The Palmetto Cluster (high performance computing cluster, Clemson University, Clemson, South Carolina, U.S.A.) to assess support for each node using 1000 replicates with 1 additional sequence replicate for each node.

Bayesian and maximum parsimony analyses for the nucleotide matrix were also conducted. Bayesian analyses were performed using Metropolis-coupled Markov chain Monte Carlo methods implemented in MrBayes 3.1.2 [52,53] using the best fit GTR+I+G substitution model without fixed parameters and considering the positions in each codon differently. The chain was run for 2.5 million generations starting from random trees, with trees sampled every 500 generations. The first 1250 trees were discarded as "burn-in" to estimate the consensus topologies and the posterior distribution of trees was used to calculate posterior probabilities for clades. Four chains were run, with one chain heated at the setting of 0.1. The Bayesian-based tree was rooted using the AGL28 sequence because MrBayes only allows one taxa as an outgroup. Maximum parsimony analyses were conducted using PAUP* 4.0b10 [49]. Maximum parsimony trees were estimated using heuristic searches, and the TBR branch swapping algorithm with 1000 random stepwise taxon additions. A total of 965 trees were obtained. Bootstrap resampling [51] was performed to assess support for each node using 1000 replicates with 10 additional sequence replicates for each node.

The duplication history of DAM genes was examined using the estimated best fit phylogenetic trees containing full length genes. Nucleotide sequences of peach DAM genes included M, I, K and C domains and were aligned as explained above (pairwise alignment parameters for gap opening 22.5 and for gap extend 0.45, multiple alignment parameters for gap opening 12.5 and for gap extend 0.25). One poplar MADS-box gene from the *SVP*/StMADS11 clade was included as the outgroup (PtMADS27). The alignment (of translated nucleotides) is presented in Figure 2. Maximum likelihood analyses, nucleotide substitution model selection and bootstrap resampling of the nucleotide distance matrix were conducted as above. The most conservative substitution model that best fits these loci as tested using Modeltest 3.7 [50] according to three sets of criteria (hLRTs, AICc and BIC) was HKY.

Bayesian and maximum parsimony analyses for the nucleotide matrix of PpDAM sequences were also conducted. Bayesian analysis was performed as above using HKY as the nucleotide substitution model. The chain was run for 10,000 generations starting from random trees, with trees sampled every 10 generations. The first 250 trees were discarded as "burn-in" to estimate the consensus topologies and the posterior distribution of trees was used to calculate posterior probabilities for clades. Four chains were run, with one chain heated at the default setting of 0.2. Maximum parsimony analysis and bootstrap resampling was conducted as above, obtaining one more parsimonious tree.

### Molecular evolutionary analyses

Sequence alignments and estimated best fit phylogenetic trees were used to test for sequence substitution patterns consistent with models of non-neutral sequence evolution. Pairwise synonymous (dS) and non-synonymous (dN) nucleotide substitution rates were estimated using the Nei-Gojobori method [54] and were performed in MEGA 4 [55].

The program CODEML from PAML 4 [56] was used to test whether sequence substitution patterns indicate significant variation of evolutionary rates among sequences (branches) or codon sites within the sequences for both trees. Significantly different ω (dS/dN rate ratio) of different branches was tested by comparing a free-ratio model (model = 1) vs. a model with a mean ratio for all lineages (model = 0). Site-specific selection was investigated by comparing the models M3, M2a and M8 vs. the null models M0, M1a, and M7, respectively, where M3, M2a and M8 can accommodate positively selected sites. Likelihood ratio tests (LRT) of different models were used to find the best fit model for the data. Statistical significance was evaluated by comparing twice the log likelihood difference between models to a χ^2 ^statistic with the degrees of freedom equal to the difference in number of parameters between models.

Variation in selection pressure among specific branches of the *SVP*/StMADS11 clade was examined for statistically significant deviations from the alternative model of no variation among branches. The branch-site model was used to test for this deviation from non-variance in substitution rates among branches [57] for the following clades: ancestral of *SVP*/StMADS11 clade and ancestral of PpDAM clade. Patterns consistent with natural selection were investigated by comparing the following models: A1 vs. the null model M0, A1 vs. the null model A2, and B vs. the null model M3 with only two site classes (k = 2).

### Ancestral reconstruction

The marginal ancestral reconstruction of progenitor peach DAM genes was estimated using CODEML. For model M8, a likelihood-based method was employed to compare the probabilities of different character assignments to an interior node at a site, and to select the character with the highest posterior probability [58].

## Authors' contributions

SJ carried out the phylogenetic and molecular evolution analysis, and drafted the manuscript. ALR participated in the design of the study and helped to draft the manuscript. GLR and AGA assisted in the analysis of the results and drafting of the manuscript. DBG conceived of the study, participated in its design and assisted in the drafting of the manuscript. All authors read and approved the final manuscript.

## Supplementary Material

Additional file 1 **Bayesian tree of 39 *Arabidopsis*, 6 peach and 48 poplar MIKC^C^-type MADS-box genes**. The tree was constructed using nucleotide sequences considering the positions in each codon differently and an HKY evolution model. The AGL28 sequence was used as the outgroup. The numbers for each interior branch indicate Bayesian posterior probabilities. Branches with less than 50% bootstrap support are collapsed. Branch lengths are proportional to the number of nucleotide changes. Click here for file

Additional file 2 **Maximum parsimony tree of 39 *Arabidopsis*, 6 peach and 48 poplar MIKC^C^-type MADS-box genes**. The tree was constructed using nucleotide sequences. The AGL28 and PtMADS63 sequences were used as outgroups. The numbers for each interior branch indicate bootstrap support of 1000 replicates. Branches with less than 50% bootstrap support are collapsed. Branch lengths are proportional to the number of nucleotide changes. Click here for file

Additional file 3 **Maximum parsimony rooted tree of 6 peach MIKC^C^-type MADS-box genes**. The tree was constructed using nucleotide sequences. The PtMADS27 sequence was used as the outgroup. The numbers for each interior branch indicate bootstrap support of 1000 replicates. Branch lengths are proportional to the number of nucleotide changes. Click here for file

Additional file 4 **Alignment of the translations of the MIKC^C^-type MADS-box genes of *Arabidopsis*, poplar and peach.** Click here for file
