# Supplementary figures and images for: Phylogenetic analysis and molecular evolution of the dormancy associated MADS-box genes from peach
Source: BMC Plant Biol. 2009 Jun 27;9:81. doi: 10.1186/1471-2229-9-81 (PMC2713236; doi:10.1186/1471-2229-9-81)

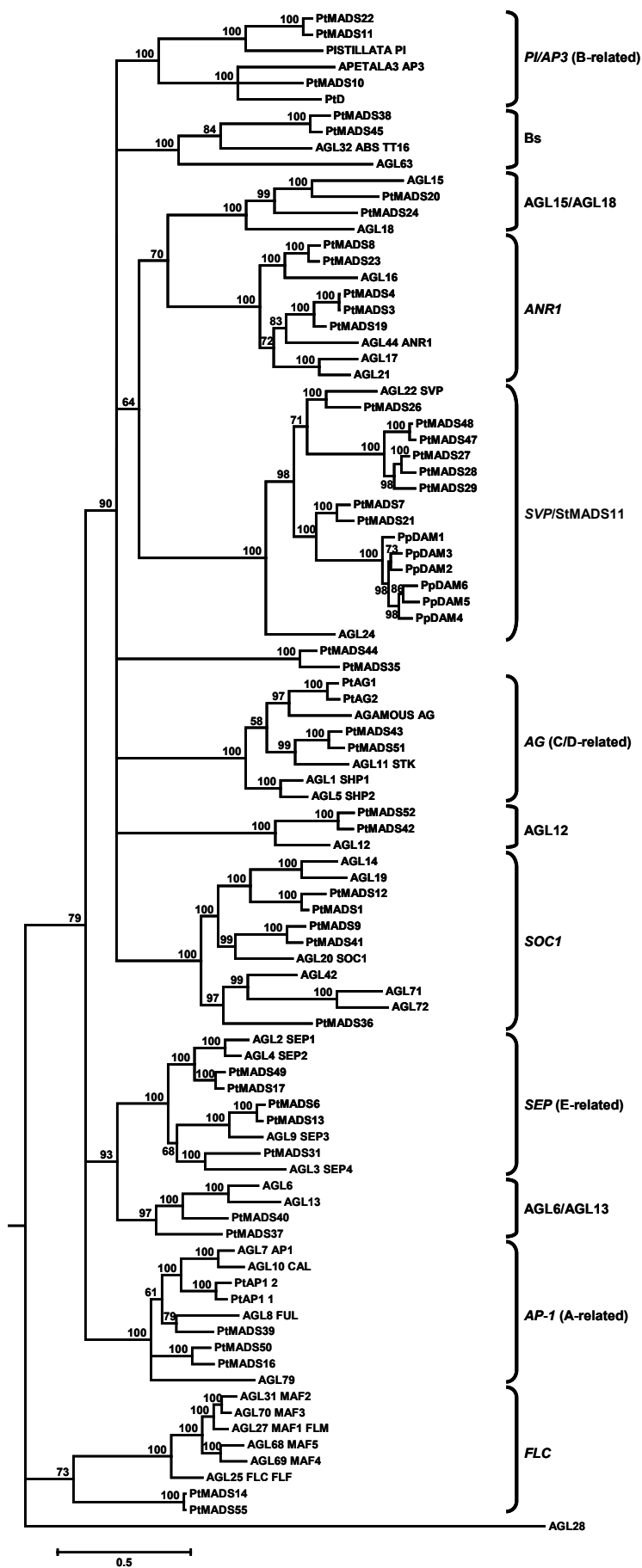

Supplement: Additional file 1 — Bayesian tree of 39 Arabidopsis, 6 peach and 48 poplar MIKCC-type MADS-box genes. The tree was constructed using nucleotide sequences considering the positions in each codon differently and an HKY evolution model. The AGL28 sequence was used as the outgroup. The numbers for each interior branch indicate Bayesian posterior probabilities. Branches with less than 50% bootstrap support are collapsed. Branch lengths are proportional to the number of nucleotide changes. [file 1471-2229-9-81-S1.pdf]

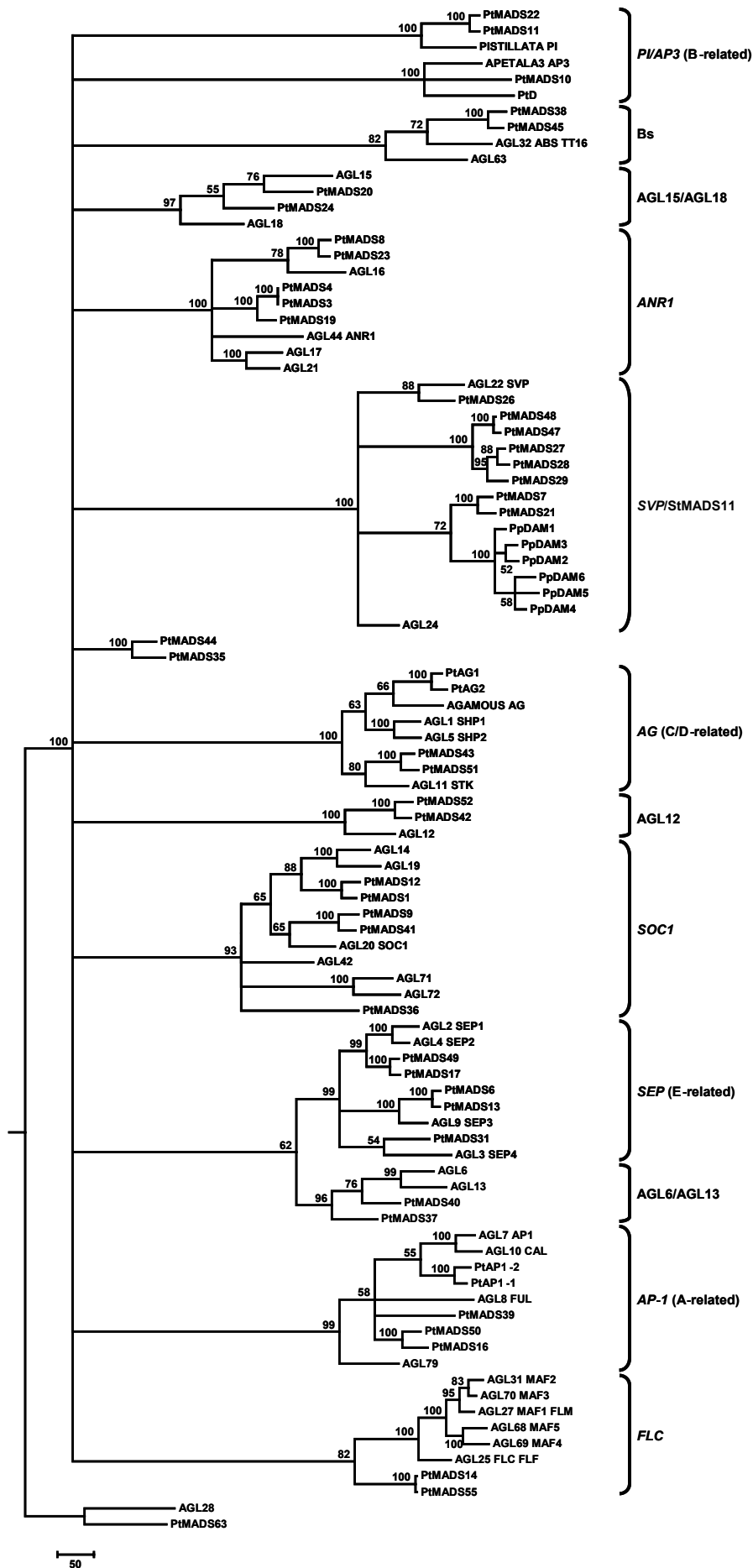

Supplement: Additional file 2 — Maximum parsimony tree of 39 Arabidopsis, 6 peach and 48 poplar MIKCC-type MADS-box genes. The tree was constructed using nucleotide sequences. The AGL28 and PtMADS63 sequences were used as outgroups. The numbers for each interior branch indicate bootstrap support of 1000 replicates. Branches with less than 50% bootstrap support are collapsed. Branch lengths are proportional to the number of nucleotide changes. [file 1471-2229-9-81-S2.pdf]

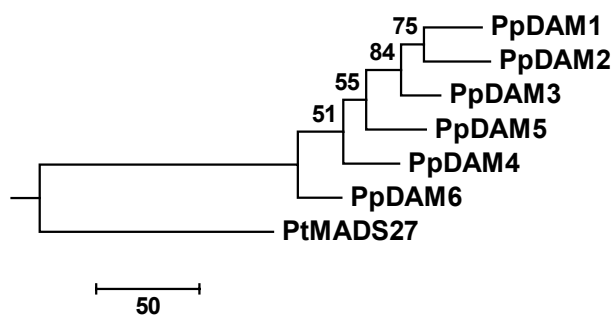

Supplement: Additional file 3 — Maximum parsimony rooted tree of 6 peach MIKCC-type MADS-box genes. The tree was constructed using nucleotide sequences. The PtMADS27 sequence was used as the outgroup. The numbers for each interior branch indicate bootstrap support of 1000 replicates. Branch lengths are proportional to the number of nucleotide changes. [file 1471-2229-9-81-S3.pdf]
