# Supplementary material for: Phylogenetic analysis and molecular evolution of the dormancy associated MADS-box genes from peach
Source: BMC Plant Biol. 2009 Jun 27;9:81. doi: 10.1186/1471-2229-9-81 (PMC2713236; doi:10.1186/1471-2229-9-81)
Supplement: Additional file 4 — Alignment of the translations of the MIKCC-type MADS-box genes of Arabidopsis, poplar and peach. [file 1471-2229-9-81-S4.pdf]

```
                        10         20         30         40         50         60         70         80         90        100
               ....|....| ....|....| ....|....| ....|....| ....|....| ....|....| ....|....| ....|....| ....|....| ....|....|
PtMADS22       MGRGKIEIKR IENASNRQVT YSKRKNGIIK KAKEITVLCD AQVSLVIFAS SGRMHEYCSP STTVVDLLDK YQKQS----- --------GK RLWDAKH---
PtMADS11       MGRGKIEIKR IENSSNRQVT YSKRRSGIIK KAKEITVLCD AQVSLVIFAS SGRMHEYCSP STTVVDLLDK YHKQS----- --------GK RLWDAKH---
PISTILLATA PI  MGRGKIEIKR IENANNRVVT FSKRRNGLVK KAKEITVLCD AKVALIIFAS NGKMIDYCCP SMDLGAMLDQ YQKLS----- --------GK KLWDAKH---
PtMADS38       MGRGKIAITR IENRTARQVT FSKRRGGLFK KTHELSVLCD AEIGLIIFSS NGKLYEFCNE SSSIPHIIRR YEISK----- --------GM RVLESND---
PtMADS45       MARGKIAITR IENRTARQVT FSKRRVGLFK KTHELSVLCD AEIGLIVFSS NGKLSEFCSE SSSIPHIIKR YEISK----- --------GM RVSESND---
AGL32 ABS TT16 MGRGKIEIKK IENQTARQVT FSKRRTGLIK KTRELSILCD AHIGLIVFSA TGKLSEFCSE QNRMPQLIDR YLHTN----- --------GL RLPDHHDD--
AGL63          MRKGKRVIKK IEEKIKRQVT FAKRKKSLIK KAYELSVLCD VHLGLIIFSH SNRLYDFCSN STSMENLIMR YQKEKEG--- --------QT TAEHSFHS--
APETALA3 AP3   MARGKIQIKR IENQTNRQVT YSKRRNGLFK KAHELTVLCD ARVSIIMFSS SNKLHEYISP NTTTKEIVDL YQTIS----- --------DV DVWATQY---
PtMADS10       MARGKIQIKK IENSTNRQVT YSKRRNGLFK KAHELTVLCD AEVSLVMVSC TDKVHDYTSP STTTKRIFDQ YQQTK----- --------GI DLWSSHY---
PtD            MGRGKIEIKK IENPTNRQVT YSKRRNGIFK KAQELTVLCD AKVSLIMFSN TNKLNEYISP STSTKKIYDQ YQNAL----- --------GI DLWGTQY---
PtMADS44       MGRGKVELKR IENSASRQVT FSKRRNGLLK KAFELSILCE AEVSLIIFSP SGKFYQFSS- -HDMERSVAR YRSEVGLPG- -------TND QRSRSLEFW-
PtMADS35       MGRGKVELKR IENPTRRQVT FSKRRNGLLK KAFELSILCD AEVSLIVFSP TGKFYQFAS- -HEMERTIAR YRSEAGLSG- -------PND SHTRSLEFW-
AGL15          MGRGKIEIKR IENANSRQVT FSKRRSGLLK KARELSVLCD AEVAVIVFSK SGKLFEYSS- -TGMKQTLSR YGNHQSSS-- -------ASK AEEDCAEV--
PtMADS20       MGRGKIEIKK IENTNSRQVT FSKRRAGLLK KAQELAILCD AEVAVIVFSN TGKLFEFSS- -SGMKRTLSR YNKFLDSPE- -------QPK IEYKAEVV--
AGL18          MGRGRIEIKK IENINSRQVT FSKRRNGLIK KAKELSILCD AEVALIIFSS TGKIYDFSS- -VCMEQILSR YGYTTASTE- -------HKQ QREHQLLICA
PtMADS24       MGRGKIEIKR IENLNSRQVT FSKRRNGLLK KARELSVLCD AEVAVIVFSS TGKLYEFSS- -TSMEHTLSR YGSGLDLDY- -------NDH PSDDHG---A
PtMADS8        MGRGKIVIRR IDNSTSRQVT FSKRRNGLLK KAKELAILCD AEVGVMIFSS TGKLYDFSS- -TSMKSVIER YNKSKDEHH- -------QMG NPTSEVKFW-
PtMADS23       MGRGKIEIRR IDNSTSRQVT FSKRRGGLLK KAKELAILCD AEVGVMIFSS TGKLYDFSS- -TSMKSVIER YNKSKEVHH- -------LMG NPTSELKFW-
PtMADS4        MGRGKIVIRR IDNSTSRQVT FSKRRSGLLK KAKELAVLCD AEVGVIVFSS TGKLYDHAN- -TSMKSIIER YSKQKEECQ- -------PLL NPASEVKLW-
PtMADS3        MGRGKIVIRR IDNSTSRQVT FSKRRSGLLK KAKELAVLCD AEVGVIVFSS TGKLYDHAN- -TSMKSIIER YSKQKEECQ- -------PLL NPASEVKLW-
PtMADS19       MGRGKIVIRR IDNSTSRQVT FSKRRNGLLK KAKELSILCD AEIGVIIFSS TGKLYDYAN- -TSMKSIIDR YNKQKEEQQ- -------QLL NPASEVKFW-
AGL16          MGRGKIAIKR INNSTSRQVT FSKRRNGLLK KAKELAILCD AEVGVIIFSS TGRLYDFSS- -SSMKSVIER YSDAKGETS- -------SEN DPASEIQFW-
AGL17          MGRGKIVIQK IDDSTSRQVT FSKRRKGLIK KAKELAILCD AEVCLIIFSN TDKLYDFAS- -SSVKSTIER FNTAKMEEQ- -------ELM NPASEVKFW-
AGL21          MGRGKIVIQR IDDSTSRQVT FSKRRKGLIK KAKELAILCD AEVGLIIFSS TGKLYDFAS- -SSMKSVIDR YNKSKIEQQ- -------QLL NPASEVKFW-
AGL44 ANR1     MGRGKIVIRR IDNSTSRQVT FSKRRSGLLK KAKELSILCD AEVGVIIFSS TGKLYDYASN -SSMKTIIER YNRVKEEQH- -------QLL NHASEIKFW-
PtAG1          LGRGKVEIKR IENTTNRQVT FCKRRSGLLK KAYELSVLCD AEVALIVFSS RGRLYEYSND --SVKSTIER YKKASAD--- --------SS NTGSVSEAN-
PtAG2          LGRGKVEIKR IENTTNRQVT FCKRRNGLLK KAYELSVLCD AEVALIVFSS RGRLYEYSNN --SVKSTIER YKKACAD--- --------SS NNGSVSEAN-
AGAMOUS AG     SGRGKIEIKR IENTTNRQVT FCKRRNGLLK KAYELSVLCD AEVALIVFSS RGRLYEYSNN --SVKGTIER YKKAISD--- --------NS NTGSVAEIN-
PtMADS43       MGRGKIEIKR IENTTNRQVT FCKRRNGLLK KAYELSVLCD AEVALIVFSS RGRLYEYANN --NIRSTIDR YKKASSD--- --------SS NASSITEIN-
PtMADS51       MGRGKIEIKR IENTTNRQVT FCKRRNGLLK KAYELSVLCD AEVSLIVFSS RGRLYEYANN --NIRSTIDR YKKVSSD--- --------SS NTASITEIN-
AGL11 STK      MGRGKIEIKR IENSTNRQVT FCKRRNGLLK KAYELSVLCD AEVALIVFST RGRLYEYANN --NIRSTIER YKKACSD--- --------ST NTSTVQEIN-
AGL1 SHP1      LGRGKIEIKR IENTTNRQVT FCKRRNGLLK KAYELSVLCD AEVALVIFST RGRLYEYANN --SVRGTIER YKKACSD--- --------AV NPPSVTEAN-
AGL5 SHP2      IGRGKIEIKR IENTTNRQVT FCKRRNGLLK KAYELSVLCD AEVALVIFST RGRLYEYANN --SVRGTIER YKKACSD--- --------AV NPPTITEAN-
PtMADS52       MARGKVQMKR IENSVHRQVT FCKRRSGLLK KAKELSVLCD AEIGVFIFSA HGKLYELATK G-TMQGLIER YMKSSRG--- --------TQ PEPAAIET--
PtMADS42       MARGKVQLRR IENHVHRQVT FCKRRAGLLK KAKELSVLCD AEIGVVIFSA HGKLYELATK G-TMQGLIER YMKSSRG--- --------AQ PEPAAMET--
AGL12          MARGKIQLKR IENPVHRQVT FCKRRTGLLK KAKELSVLCD AEIGVVIFSP QGKLFELATK G-TMEGMIDK YMKCTGG--- --------GR GSSSATFTAQ
AGL2 SEP1      MGRGRVELKR IENKINRQVT FAKRRNGLLK KAYELSVLCD AEVALIIFSN RGKLYEFCSS -SNMLKTLDR YQKCSYG--- ------SIEV NNKPAKEL--
AGL4 SEP2      MGRGRVELKR IENKINRQVT FAKRRNGLLK KAYELSVLCD AEVSLIVFSN RGKLYEFCST -SNMLKTLER YQKCSYG--- ------SIEV NNKPAKEL--
PtMADS49       MGRGRVELKR IENKINRQVT FAKRRNGLLK KAYELSVLCE AEVALIIFSN RGKLFEFCST -SNMLKTLER YQKCSYG--- ------AEEV N-KPAKEL--
PtMADS17       MGRGRVELKR IENKINRQVT FAKRRNGLLK KAYELSVLCD AEVALIIFSN RGKLYEFCST -SNMLKTLER YQKCSYG--- ------AEEV N-KPAKEL--
PtMADS6        MGRGRVELKR IENKINRQVT FAKRRNGLLK KAYELSVLCD AEVALIIFSN RGKLYEFCSS -SSMLKTLER YQKCNYG--- ------APEP NVSAREAL--
PtMADS13       MGRGRVELKR IENKINRQVT FAKRRNGLLK KAYELSVLCD AEIALIIFSN RGKLYEFCSS -SSMLKTLER YQKCNYG--- ------APEP NVSAREAL--
AGL9 SEP3      MGRGRVELKR IENKINRQVT FAKRRNGLLK KAYELSVLCD AEVALIIFSN RGKLYEFCSS -SSMLRTLER YQKCNYG--- ------APEP NVPSREALA-
PtMADS31       MGRGRVELKR IENKINRQVT FAKRRNGLLK KAYELSVLCD AEVALIIFSN SGKLFEFCSS -SNMATTIEK YQRFSYG--- ------ALEG GQSEKETQ--
AGL3 SEP4      MGRGKVELKR IENKINRQVT FAKRRNGLLK KAYELSVLCD AEIALLIFSN RGKLYEFCSS PSGMARTVDK YRKHSYA--- ------TMDP NQSAKDLQ--
AGL6           MGRGRVEMKR IENKINRQVT FSKRRNGLLK KAYELSVLCD AEVALIIFSS RGKLYEFGSV --GIESTIER YNRCYNC--- ------SLSN N-KPEETT--
AGL13          MGRGKVEVKR IENKITRQVT FSKRKSGLLK KAYELSVLCD AEVSLIIFST GGKLYEFSNV --GVGRTIER YYRCKDN--- ------LLDN D-TLEDT---
PtMADS40       MGRGRVELKR IENKINRQVT FSKRRNGLLK KAYELSVLCD AEVALIIFSS RGKLYEFGSA --SVTKTLER YQRCCYT--- ------PQEN S-IERET---
PtMADS37       MGRGKVVLER IENKISRQVT FSKRRNGLLK KAYELSLLCD AEVALIIFSS HGKLFEFSSI --DMNSILQR YRQCCYS--- ------TQDT N-IPEEGS--
AGL7 AP1       MGRGRVQLKR IENKINRQVT FSKRRAGLLK KAHEISVLCD AEVALVVFSH KGKLFEYSTD -SCMEKILER YERYSYA--- ------ERQL IAPESDVN--
AGL10 CAL      MGRGRVELKR IENKINRQVT FSKRRTGLLK KAQEISVLCD AEVSLIVFSH KGKLFEYSSE -SCMEKVLER YERYSYA--- ------ERQL IAPDSHVNA-
PtAP1-2        MGRGRVQLKR IENKINRQVT FSKRRTGLLK KANEISVLCD AEVALIVFSH KGKLFEYSTD -DSMEKILER YERYSYA--- ------ERQL VATDLDSQ--
PtAP1-1        MGRGRVQLKR IENKINRQVT FSKRRTGLLK KAHEISVLCD AEVALIVFSH KGKLFEYSTN -ACMEKILER HERYSYA--- ------ERQL VATDLDSQ--
AGL8 FUL       MGRGRVQLKR IENKINRQVT FSKRRSGLLK KAHEISVLCD AEVALIVFSS KGKLFEYSTD -SCMERILER YDRYLYS--- ------DKQL VGRDVSQS--
PtMADS39       MGRGRIQLKR IENKINRQVT FSKRRSGLLK KAHEISVLCD AEVALIVFST KGKLFEYATD -SCMERILER YERYSYA--- ------ERQL LANDDPEN--
PtMADS50       MGRGRVQLKR IENNISRQVT FSKRRTGLLK KAHEISVLCD ADVAVIVFST KGKLFEYSTD -SSMESILER YERCSYA--- ------EQQF VPHG-PEH--
PtMADS16       MGRGRVQLKR IENKINRQVT FSKRRTGLLK KAHEISVLCD ADVALIVFST RGKLFEYSTD -SSMESILER YERCSYL--- ------EQQL VPNG-SEH--
AGL79          MGRGRVQLRR IENKIRRQVT FSKRRTGLVK KAQEISVLCD AEVALIVFSP KGKLFEYSAG -SSMERILDR YERSAYA--- ------GQDI PTPNLDSQ--
AGL31 MAF2     MGRKKVEIKR IENKSSRQVT FSKRRNGLIE KARQLSILCE SSIAVLVVSG SGKLYKSASG -DNMSKIIDR YEIHHAD--- --------EL EALDLAEK--
AGL70 MAF3     MGRRKVEIKR IENKSSRQVT FSKRRKGLIE KARQLSILCE SSIAVVAVSG SGKLYDSASG -DNMSKIIDR YEIHHAD--- --------EL KALDLAEK--
AGL27 MAF1 FLM MGRRKIEIKR IENKSSRQVT FSKRRNGLID KARQLSILCE SSVAVVVVSA SGKLYDSSSG -DDISKIIDR YEIQHAD--- --------EL RALDLEEK--
AGL68 MAF5     MGRRRVEIKR IENKSSRQVT FCKRRNGLME KARQLSILCG SSVALFIVSS TGKLYNSSSG -DSMAKIISR FKIQQAD--- --------DP ETLDLEDK--
AGL69 MAF4     MGRRKVEIKR IENKSSRQVT FCKRRNGLME KARQLSILCE SSVALIIISA TGRLYSFSSG -DSMAKILSR YELEQAD--- --------DL KTLDLEEK--
AGL25 FLC FLF  MGRKKLEIKR IENKSSRQVT FSKRRNGLIE KARQLSVLCD ASVALLVVSA SGKLYSFSSG -DNLVKILDR YGKQHAD--- --------DL KALDHQSK--
PtMADS14       MGRKKVELKR IEKKICRQIT FSKRRNGLIK KARDLSLLCD VQVALLVFSS SGKLYEFSSA -GSLAKILKR HGSYFEEKTA LSNGANDAEL YHGKYEKK--
PtMADS55       MGRKKVELKR IEKKICRQIT FSKRRNGLIK KARDLSLLCD VQVALLVFSS SGKLYEFSSA -GSLAKILKR HRSYFEEKTA LSNGANDAEL YHGKYEKK--
AGL14          MVRGKTEMKR IENATSRQVT FSKRRNGLLK KAFELSVLCD AEVALIIFSP RGKLYEFSSS -SSIPKTVER YQKRIQD--- -------LGS N-HKR-NDN-
AGL19          MVRGKTEMKR IENATSRQVT FSKRRNGLLK KAFELSVLCD AEVALVIFSP RSKLYEFSSS -S-IAATIER YQRRIKE--- -------IGN N-HKR-NDN-
PtMADS12       MVRGKTQMKR IENATSRQVT FSKRRNGLLK KAFELSVLCD AEVALIVFSS RGKLYEFSSS -S-INRTIER YQKRAKD--- -------VGI S-SKMVQDN-
PtMADS1        MVRGKTQMKR IENATSRQVT FSKRRNGLLK KAFELSVLCD AEVALIVFST RGKLYEFSSS -S-MNRTIES YQKRAKD--- -------VGT S-SKMVKDN-
PtMADS9        MVRGKTQMRR IENATSRQVT FSKRRNGLLK KAFELSVLCD AEVAVIVFSP RGKLYEFGSS -S-VQETIER YQRHVKE--- -------SNT N-KQTSELN-
PtMADS41       MVRGKTQMRR IENATSRQVT FSKRRNGLLK KAFELSVLCD AEVALIVFSP RGKLYEFAST -S-MQETIER YRRHVKE--- -------NNT N-KQPVEQN-
AGL20 SOC1     MVRGKTQMKR IENATSRQVT FSKRRNGLLK KAFELSVLCD AEVSLIIFSP KGKLYEFASS -N-MQDTIDR YLRHTKD--- -------RVS T-KPVSEEN-
AGL42          MVRGKIEMKK IENATSRQVT FSKRRNGLLK KAYELSVLCD AQLSLIIFSQ RGRLYEFSSS -D-MQKTIER YRKYTKD--- -------HET S-NHDSQIH-
PtMADS36       MARGKVQLKR IENATSRQVT FSKRKNGLLK KAYELSILCD AEVAVIIFSQ KGTLFKFASI -DQIQKTIDR YRKNAKQ--- -------LHT D-RIDVEQS-
AGL71          MVRGKIEIKK IENVTSRQVT FSKRRSGLFK KAHELSVLCD AQVAAIVFSQ SGRLHEYSSS -Q-MEKIIDR YGKFSNA--- -------FYV AERPQVERY-
AGL72          MVRGKIEIKK IENVTSRQVT FSKRRSGLFK KAHELSVLCD AQVAAMIFSQ KGRLYEFASS -D-IRNTIKR YAEYKRE--- -------YFV AETHPIEQY-
AGL22 SVP      MAREKIQIRK IDNATARQVT FSKRRRGLFK KAEELSVLCD ADVALIIFSS TGKLFEFCSS --SMKEVLER HNLQSKNLE- -------KLD QPSLELQLV-
PtMADS26       MARERIQIKK IDNATARQVT FSKRRRGLFK KAEELSVLCD ADVALIIFSS TGKLFEFSSS --SMKEILER HNLHSKNLE- -------KLE QPSLELQLV-
PtMADS7        MAREKIKIKK IDNVTARQVT FSKRRRGLFK KAEELSVLCD AEVAVIIFSA TGKLFEYSSS --SMKDVLAR YNLHSNNLD- -------KIN PPSLELQLE-
PtMADS21       MAREKIKIKK IDNVAARQVT FSKRRRGLLK KAEELSVLCD VEVAVIIFSA TGKLFEYSSS --SMKDVLAR YNLHSNNLD- -------KLN QPSLELQLE-
AGL24          MAREKIRIKK IDNITARQVT FSKRRRGIFK KADELSVLCD ADVALIIFSA TGKLFEFSSS --RMRDILGR YSLHASNIN- -------KLM DPPSTHLRL-
PtMADS48       MTRKKIPIKK IDNTTARQVS FSKRRRGLFK KACELSILCD AEIALMVFSA TGKFFEYSNS --SIGQVIER RNLHPKNLD- -------KFS QPSVELQLD-
PtMADS47       MARKKIQIKK IDNTSARQVS FSKRRRGLFK KAFELSILCD AEIALMVFSA TGKFFEYSNS --SIGQVIER RNLHPKNLD- -------TFS QPSVELQLD-
PtMADS27       MTRKKIQIKK IDNTAARQVT FSKRRRGLFK KAYELSTLCD AEIALMVFSA TGKLFEYSNS --SMGQVIER RNLHPKNIN- -------TLD QPSLEKQLD-
PtMADS28       MTRKKIQIKK IDNTAARQVT FSKRRRGLFK KAYELSTLCD AEIALTVFSA TGKLFEYSNT ---------- ---------- ---------- -----RELD-
PtMADS29       MTRRKIQIKK IDDTIARQVT FSKRRGGLFK KAYELSTLCD AEIALMVFSA SGKLYEYSNS --SMGQVIEK RNLHPKNID- -------MFG QPSLELQPD-
PpDAM1         MTREKIKIKK IDNLPARQVT FSKRRRGIFK KAAELSVLCE SEVAVVIFSA TGKLFDYSSS --SMKDVIER YQEHINGAE- -------KFD EPSIELQPE-
PpDAM3         MMRKKIKIKK IDYLPARQVT FSKRRRGIFK KAAELSVLCE SKVAVVIFSA TGKLFDYSSS --SIKDVIER YKAHTNGVE- -------KSD KPSVELQLE-
PpDAM2         TMRKKIKIKK IDYLPARQVT FSKRRRGIFK KAEELSVLCE SEVAVVIFSA TGKLFDYSSS --STKDVVER YQAHTNGVE- -------KSD EPSVELQLE-
PpDAM6         MMREKIKIKK IDYLPARQVT FSKRRRGLFK KAAELSVLCE SEVAVIIFSA TDKLFDYSSS --STEDVIER YKAHTNDLE- -------KSN KQFLELQLE-
PpDAM5         MMRNKIKIKK IDYLPARQVT FSKRRRGLFK KAAELSVLCE SEVAVVIFSA TGKLFDYSSS --STKDVIER YNADINGVE- -------KLN NQEIELQLE-
PpDAM4         MMREKIKIKK IDYLPARQVT FSKRRRGIFK KAAELSVLCE SEVAVVIFSA TGKLFDYSSS --SIKDVIER YEVRTNGVE- -------KSD EQSLELQLE-
AGL28          LGRRKIELVK MTNESNLQVT FSKRRSGLFK KGSELCTLCD AEIAIIVFSP SGKAYSFGHP --NVNKLLDH SLGRVIR--- -------HNN --TNFAES--
PtMADS63       RGRQKVEMVK MSKESNLQVT FSKRRSGLFK KASELSTLCG AEIAIIVFSP GNRVFSFGHP --GVETVIDR YFTRNPP--- -------QNS GTMQLIEA--
```

```
                       110        120        130        140        150        160        170        180        190
                ....|....| ....|....| ....|....| ....|....| ....|....| ....|....| ....|....| ....|....| ....|....| ...
PtMADS22        -------ENL SKEIDRIKKE NDSMQIELR- HLKGEDISSL HHTELMAIEE ALDAGLAAVC KKQ----MEY HSMLEQNEKM LDEEFKRLQF VLQ
PtMADS11        -------ENL SNEIDRIKKE NESMQIELR- HLKGQDISSL PHKELMAIEE ALDTGLAAVR KKQ----MEF HSMLEQNEKI LDEEFKHLQF VLQ
PISTILLATA PI   -------ENL SNEIDRIKKE NDSLQLELR- HLKGEDIQSL NLKNLMAVEH AIEHGLDKVR DHQ----MEI LISKRRNEKM MAEEQRQLTF QLQ
PtMADS38        ------WEQI QKESKRIRKE TDDLQLSVR- CYKGENLSSL HHEGLVELEK QLECSVNKVR AQKLELLQQQ VDNLRRKEKM LEEENQQIQY HLH
PtMADS45        ------SEQI LKELKRIRKE TDDLQLSMR- CYKGESLSSL HYEDLVELEK QLECSVNKVR ARKFELLQQQ VDNLRRKEKM LEVENQQIQY HLV
AGL32 ABS TT16  ------QEQL HHEMELLRRE TCNLELRLR- PFHGHDLASI PPNELDGLER QLEHSVLKVR ERK-----QQ LENLSRKRRM LEEDNNNMYR WLH
AGL63           ------CSDC VKTKESMMRE IENLKLNLQ- LYDGHGLNLL TYDELLSFEL HLESSLQHAR ARKSE-FMHQ QQQQQTDQKL KGKEKGQGSS WEQ
APETALA3 AP3    -------ERM QETKRKLLET NRNLRTQIK- QRLGECLDEL DIQELRRLED EMENTFKLVR ERKFKSLGNQ IETTKKKNKS QQDIQKNLIH ELE
PtMADS10        -------EIM KENLEKLKEV NMKIRREMR- QRMGQCLNGL SFQDLQSLES DMESAWRVIH DRADRVLTNQ IETSKKKARN VEQINRKLQV ELE
PtD             -------EKM QEHLRKLNDI NHKLRQEIR- QRRGEGLNDL SIDHLRGLEQ HMTEALNGVR GRKYHVIKTQ NETYRKKVKN LEERHGNLLM EYE
PtMADS44        ---------- RCEIEELRRT ITKTEAQLR- HFIGEDIAPL GLKELKQLER QLKTGVERIR SKKKRVISEH IKLLKSEQRA LQEENARLQK RLH
PtMADS35        ---------- RREIEELQKT INETEAKLR- HCIGEDIEML GMKELKQLER QLKAGVERVR SKKLRIAAEH VNWLKGKQRS IQEENACLKK RVR
AGL15           ---------- ----DILKDQ LSKLQEKHL- QLQGKGLNPL TFKELQSLEQ QLYHALITVR ERKERLLTNQ LEESRLKEQR AELENETLRR QV-
PtMADS20        ---------- ----DVLKEE IAKLQVKQL- RLSGMDLTGL SLKELQQLEN QLNEGLLFVK EKKEHLLMEQ LEQSRVQEQR AMLENETLRR QAR
AGL18           SHG---NEAV LRNDDSMKGE LERLQLAIE- RLKGKELEGM SFPDLISLEN QLNESLHSVK DQKTQILLNQ IERSRIQEKK ALEENQILRK QVE
PtMADS24        EHS---NSAE VN---AVKDE LSKLRLTCL- QMMGQQLDGL SFKELQHLEH QLSAGILSVK DKKEQMLMDQ LKKSKMQEQK ATLENESLRK QIE
PtMADS8         ---------- QREAAVLRQQ LQTLQENHR- QMMGEQLSGL SVTDLQNLES QLEMSLQGVR MKKDQILMDQ IQELNRKGNL IHQENVELY- ---
PtMADS23        ---------- QRETAMLRQQ LQNLQENHR- QMMGEELSGL SVKDLQNLEN QLEMSLRGVR MKKDQNLMDE ILELNRKGNL IHQENMELYK KAN
PtMADS4         ---------- KREAASLGKE LQCLKEYHR- QLMGEELSGL SIKDLENLEN QLEKSMKGVR IKKEQILTDE IKEMSQKGNL IYQENLELHK KVD
PtMADS3         ---------- KREAASLGKE LQCLKEYHR- QLMGEELSGL SIKDLENLEN QLEKSMKGVR IKKEQILTDE IKEMSQKGNL IYQENLELHK KVD
PtMADS19        ---------- QREAASLRKE LQYLQECHR- QLMGEELSGL SAKDLQNLEN QLEMSLKG-- ---EQILTDE IKDLNRKGNL IYQENLELHK KVK
AGL16           ---------- QKEAAILKRQ LHNLQENHR- QMMGEELSGL SVEALQNLEN QLELSLRGVR MKKDQMLIEE IQVLNREGNL VHQENLDLHK KVN
AGL17           ---------- QREAETLRQE LHSLQENYR- QLTGVELNGL SVKELQNIES QLEMSLRGIR MKREQILTNE IKELTRKRNL VHHENLELSR KVQ
AGL21           ---------- QREAAVLRQE LHALQENHR- QMMGEQLNGL SVNELNSLEN QIEISLRGIR MRKEQLLTQE IQELSQKRNL IHQENLDLSR KVQ
AGL44 ANR1      ---------- QREVASLQQQ LQYLQECHR- KLVGEELSGM NANDLQNLED QLVTSLKGVR LKKDQLMTNE IRELNRKGQI IQKENHELQN IVD
PtAG1           ------AQYY QQEAAKLRSQ IGNLQNSNRH MLGEA-LSSL SVKELKSLEI RLEKGISRIR SKKNELLFAE IEYMQKREVD LHNNNQLLRA KIS
PtAG2           ------AQFY QQEAAKLRSQ IGNLQNSNRN MLGES-LSAL SVKELKSLEI KLEKGIGRIR SKKNELLFAE IEYMQKREID LHNNNQLLRA KIA
AGAMOUS AG      ------AQYY QQESAKLRQQ IISIQNSNRQ LMGET-IGSM SPKELRNLEG RLERSITRIR SKKNELLFSE IDYMQKREVD LHNDNQILRA KIA
PtMADS43        ------AQYY QQESAKLRQQ IQMLQNSNRH LMGDA-VSNL SVKELKQLEN RLERGITRIR SKKHELLLAE IEYLQKREIE LENESVCLRT KIA
PtMADS51        ------AQYY QQESAKMRQQ IQLLQNSNRH LMGEA-VSNL SVKELKQLEN RLERGMTRIR SKKHELLLAE IEYMQKREIE LENESACLRT KIA
AGL11 STK       ------AAYY QQESAKLRQQ IQTIQNSNRN LMGDS-LSSL SVKELKQVEN RLEKAISRIR SKKHELLLVE IENAQKREIE LDNENIYLRT KVA
AGL1 SHP1       ------TQYY QQEASKLRRQ IRDIQNSNRH IVGES-LGSL NFKELKNLEG RLEKGISRVR SKKNELLVAE IEYMQKREME LQHNNMYLRA KIA
AGL5 SHP2       ------TQYY QQEASKLRRQ IRDIQNLNRH ILGES-LGSL NFKELKNLES RLEKGISRVR SKKHEMLVAE IEYMQKREIE LQNDNMYLRS KIT
PtMADS52        ----QPDMDV KEEINMLKQE IEILQKGLRY MFGGR-AAEM TLDELIELEK HLEIWIYQIR STKMDIMFKE IQQLRNKEGI LKAANQYLQD KVE
PtMADS42        ----QPAPDL --EINMLKQE IEVLQKGLRY MFGARAAAEM SLDELLVLEK HLEIWIYQIR STKMDIMFKE IQQLRNKEEI LTAANQHLHN KVE
AGL12           EQLQPPNLDP KDEINVLKQE IEMLQKGISY MFGGG-DGAM NLEELLLLEK HLEYWISQIR SAKMDVMLQE IQSLRNKEGV LKNTNKYLLE KIE
AGL2 SEP1       -------ENS YREYLKLKGR YENLQRQQR- NLLGEDLGPL NSKELEQLER QLDGSLKQVR SIKTQYMLDQ LSDLQNKEQM LLETNRALAM KLD
AGL4 SEP2       -------ENS YREYLKLKGR YENLQRQQR- NLLGEDLGPL NSKELEQLER QLDGSLKQVR CIKTQYMLDQ LSDLQGKEHI LLDANRALSM KLE
PtMADS49        -------ESS YREYLKVKAK FETLQRTQR- NLLGEDLGPL NTKELEQLER HLESSLKQVR STKTQYMLDQ LGDLQNKEHM LLEANRALTI KLD
PtMADS17        -------ESS YREYLKVKAR FEALQRTQR- NLLGEDLGPL NTKELEQLER QLESSLNQVR STKTQYMLDQ LADLQNKEHL LLEANRGLTI KLD
PtMADS6         ------ELSS QQEYLKLKAR YEALQRTQR- NLLGEELGPL SSKELESLER QLDMSLKQIR STRTQYMLDQ LHDLQHKEHM LTAANKSLKE RLM
PtMADS13        ------ELSS QQEYLKLKAR YEALQRTQR- NLLGEDLGPL SSKELESLER QLDMSLKQIR STRTQYMLDQ LNDLQHKEHM LTAANKSLRE RLM
AGL9 SEP3       ------ELSS QQEYLKLKER YDALQRTQR- NLLGEDLGPL STKELESLER QLDSSLKQIR ALRTQFMLDQ LNDLQSKERM LTETNKTLRL RLA
PtMADS31        -------QNN YQEYLKLKTR VDVLQRSQR- NLLGEDLGNL GTMELDQLEN QLDSSLKQIR SRKGQFVLDE LSELQRKEEL LLETNNALKR KLE
AGL3 SEP4       --------DK YQDYLKLKSR VEILQHSQR- HLLGEELSEM DVNELEHLER QVDASLRQIR STKARSMLDQ LSDLKTKEEM LLETNRDLRR KLE
AGL6            -------QSW CQEVTKLKSK YESLVRTNR- NLLGEDLGEM GVKELQALER QLEAALTATR QRKTQVMMEE MEDLRKKERQ LGDINKQLKI KFE
AGL13           -------QGL RQEVTKLKCK YESLLRTHR- NLVGEDLEGM SIKELQTLER QLEGALSATR KQKTQVMMEQ MEELRRKERE LGDINN--KL KLE
PtMADS40        -------QSW YLEATKLKAK YESLQRTQRL HLLGEDLGPL NVKELQNLEK QLEGALALAR QRKQQVLTEQ MEDLRKKERH LGDLNRHLKL KLE
PtMADS37        -------HNL YQEVSRLRAK CETLQRSQR- NFLGEDLEPL AFKELEKIEK QLDKTLSQAR QRKTQLLFDK MEELRLKEQE LEEENKQLKT KV-
AGL7 AP1        -------TNW SMEYNRLKAK IELLERNQR- HYLGEDLQAM SPKELQNLEQ QLDTALKHIR TRKNQLMYES INELQKKEKA IQEQNSMLSK Q-I
AGL10 CAL       ------QTNW SMEYSRLKAK IELLERNQR- HYLGEELEPM SLKDLQNLEQ QLETALKHIR SRKNQLMNES LNHLQRKEKE IQEENSMLTK Q-I
PtAP1-2         -------GNW TLEYNRLKAK VELLQRNHR- NYLGEDLDSM SLKELQNLEQ QIDTALKHIR ARKNHLMSQS ISELQRKEKA IQVQNNMLVK Q-K
PtAP1-1         -------GNW TLEYNRLKAK VELLQRNHR- HYLGEDLDSV SLKELQNLEQ QIDTALKLIR ERKNHLMYQS ISELQIKEKA IKEQNNMLVK Q-I
AGL8 FUL        ------E-NW VLEHAKLKAR VEVLEKNKR- NFMGEDLDSL SLKELQSLEH QLDAAIKSIR SRKNQAMFES ISALQKKDKA LQDHNNSLLK K-I
PtMADS39        ------HGSW TLEYAKLKAR VDVLQRNQR- HFMGEDLDSL NIKELQNLEH QIDSALKHVR SRKNQLMYES ISELQKKDKA LQEQNNMLAK K-V
PtMADS50        ------QGSW FLEHPKLRAR VELLQRNLR- NYTGQDLDPL SYKELQHLEQ KIDTALKSVR SRKNQLVHES LAEMQKKEKA LQDQNNILVE Q-V
PtMADS16        ------QESW SLEHPKLMAR VEILQRNLR- NYAGQELDPL SLKELQYLEQ QIDTALKRIR SRKNQLIHES LNELRKKEKE LQEQNNILAE Q-V
AGL79           -------GEC STECSKLLRM IDVLQRSLR- HLRGEEVDGL SIRDLQGVEM QLDTALKKTR SRKNQLMVES IAQLQKKEKE LKELKKQLTK K-A
AGL31 MAF2      ---------- ---TRNYLPL KELLEIVQS- KLEESNVDNA SVDTLISLEE QLETALSVTR ARKTELMMGE VKSLQKT--- ---------- ---
AGL70 MAF3      ---------- ---IRNYLPH KELLEIVQS- KLEESNVDNV SVDSLISMEE QLETALSVIR AKKTELMMED MKSLQEREKL LIEENQILAS ---
AGL27 MAF1 FLM  ---------- ---IQNYLPH KELLETVQS- KLEEPNVDNV SVDSLISLEE QLETALSVSR ARKAELMMEY IESLKEKEKL LREENQVLAS ---
AGL68 MAF5      ---------- ---TQDYLSH KELLEIVQR- KIEEAKGDNV SIESLISMEE QLKSALSVIR ARKTELLMEL VKNLQDKEKL LKEKNKVLAS ---
AGL69 MAF4      ---------- ---TLNYLSH KELLETIQC- KIEEAKSDNV SIDCLKSLEE QLKTALSVTR ARKTELMMEL VKTHQEKEKL LREENQSLTN QLI
AGL25 FLC FLF   ---------- ---ALNYGSH YELLELVDS- KLVGSNVKNV SIDALVQLEE HLETALSVTR AKKTELMLKL VENLKEKEKM LKEENQVLAS ---
PtMADS14        ---------- ---IKSFAEL LQTVQSIHR- QVGNSNFEEL TLSDLEQTEM QLDAALRRTR ARKTELMLET INALNDKEKT LREENQRLQT ---
PtMADS55        ---------- ---IKSFAEL LQTVQSIHR- QVGDSNFEEL TLSDLEQTEM QVDAALRRTR ARKTELMLET INALNDKEKT LREENQRLQT ---
AGL14           ------SQQS KDETYGLARK IEHLEISTR- KMMGEGLDAS SIEELQQLEN QLDRSLMKIR AKKYQLLREE TEKLKEKERN LIAENKMLME KCE
AGL19           ------SQQA RDETSGLTKK IEQLEISKR- KLLGEGIDAC SIEELQQLEN QLDRSLSRIR AKKYQLLREE IEKLKAEERN LVKENKDLKE KWL
PtMADS12        ------IQPV KEDTFTLAKK IELLEVSKR- KLLGEGLETC STDDLQQLEN QLGRSLTRIR ARKNQLFRER IEKLKGEEKI LLEENTRLRE KNC
PtMADS1         ------MQPV KEDAFTLAKK IELLEVSKR- KLLGDGLEPC SIDDLQQLEN QLERSLTRIR ARKNQLFREQ IEKLKGEEKI LMEENTELRE K--
PtMADS9         ------MEQL KGEAASMIKK IEILEVSKR- KLLGECLGSC TVEELQQIEQ QLERSVSTIR ARKNQVFREQ IEQLKQKEKQ LTAENARLSN KSG
PtMADS41        ------MLQL KEEAASMIKK IEHLEVSKR- KLLGECLGSC TVEELQQIEQ QLERSVSTIR ARKNQVFKEQ IELLRQKEKL LAAENARLSD E--
AGL20 SOC1      ------MQHL KYEAANMMKK IEQLEASKR- KLLGEGIGTC SIEELQQIEQ QLEKSVKCIR ARKTQVFKEQ IEQLKQKEKA LAAENEKLSE KWG
AGL42           ------LQQL KQEASHMITK IELLEFHKR- KLLGQGIASC SLEELQEIDS QLQRSLGKVR ERKAQLFKEQ LEKLKAKEKQ LLEENVKLHQ KNV
PtMADS36        ------KEQL RQESANMAKK IEIIEILQR- KLLGQDLDSC SPEELHDIDN QLEISLSNIR ARKTQLFKEQ IEQLQAKERL LLMENARLTK QCD
AGL71           ------LQEL KMEIDRMVKK IDLLEVHHR- KLLGQGLDSC SVTELQEIDT QIEKSLRIVR SRKAELYADQ LKKLKEKERE LLNERKR--L LEE
AGL72           ------VQGL KKEMVTMVKK IEVLEVHNR- KMMGQSLDSC SVKELSEIAT QIEKSLHMVR LRKAKLYEDE LQKLKAKERE LKDERVRLSL KKT
AGL22 SVP       ---------E NSDHARMSKE IADKSHRLR- QMRGEELQGL DIEELQQLEK ALETGLTRVI ETKSDKIMSE ISELQKKGMQ LMDENKR--- LRQ
PtMADS26        ---------E DSTCSRLSKE VAEKSHQLR- QMRGEDLRGL DIDELLQLEK SLEAGLSCVI EKKGEKIMNE ITDLQRKGMQ LMEENER--- LKQ
PtMADS7         ---------- NSNHMRLSKE VSEKSHQLR- RMRGEDLHGL NIEELQQLEK ALEVGLSRVL ETKGERIMNE ISTLERKGVQ LLEENKQ--- LKQ
PtMADS21        ---------- NSNHMRLRKE VSEKSHQLR- RMRGEELQGL NIEELQQLEK VLEVGLCCVL ETKGERIMNE ISTLERKGAQ LLEENKH--- LKQ
AGL24           ---------E NCNLSRLSKE VEDKTKQLR- KLRGEDLDGL NLEELQRLEK LLESGLSRVS EKKGECVMSQ IFSLEKRGSE LVDENKR--- LRD
PtMADS48        ---------- SAVHAMLNKE IAEKTRELR- RTRGEDLQGL NMEELEKLEK LIEGSLCRVM ETKGEKILKE VDALKSKEQQ LIEENQR--- LTQ
PtMADS47        ---------- SAVHAMLNKE IAEKTRELR- RTRGEDLQGL NMEELEKLEK LIEKSLCRVI ETKGEKILKE VDALKSKEHQ LIEENQR--- LKQ
PtMADS27        ---------- GGVHAMLIKE IAKKNRELR- HMRGEDLQGL DLEELQKLEK IMEGSLRRLV EEKGGKIINE IDALKTKGEQ LIEENQR--- LKQ
PtMADS28        ---------- GGVHAMLIKE IAEKNRELR- HMRGEDLQGL SLEELKKIEK LIEGSLRRVV EEKEEKSTKD INALKTKGEQ LAEENQR--- LKQ
PtMADS29        ---------- GAVYATLNKE IAEKTRELS- QVRGEDLQGL NLEELHKLEK LIETSLCRVV EEKGGKIINE INTLKNEGEQ LVEENRR--- LRQ
PpDAM1          ---------- KENHIRLSKE LEEKSRQLR- QMKGEDLEEL NFDELQKLEQ LVDASLGRVI ETKDELIMSE IMALKRKRAE LVEANKQ--- LRQ
PpDAM3          ---------- NENQIGLSKE LKEKSHQLR- QMKAEDLEEL NFDELQKLEQ LVDASLGRVI ETKEELRMSE IMALERKGAE LVEANNQ--- LRQ
PpDAM2          ---------- IENHIRLTKE LEEKSCQLR- QIKGEDLEEL NFDELQKLEQ LVDASLGRVI ETEEELIMSE IMALERKGAE LVEANNQ--- LRQ
PpDAM6          ---------- NENHIKLSKE LEEKSRQLR- QMKGEDLQGL NMDELLKLEQ LVEASLGRVI ETKEELIMSE IMALEKKGAE LVEANNQ--- LRQ
PpDAM5          ---------- NENHIKLSKE LEEKSRQLR- QMKGEDLEGL NLDELLKLEQ LVEASLGRVM ETKEELIKSE IMALERKGTE LVEANNQ--- LRQ
PpDAM4          ---------- NENHTKLSTE LEEKNRQLR- QMKGEDLEEL DLDELLKLEQ LVEATLVRVM ETKEELIMSD IVALEKKGTE LVEANNQMVM LRE
AGL28           ---------- -----RTKLR IQMLNESLTE VMAEKEKEQE TKQSIVQNER ENKDAEKWWR NSPTELNLAQ STSMKCDLEA LKKEVDEKVA QLH
PtMADS63        ---------- -----HRNAT VRELNMQLTQ VVNQFEIEKK RGEELSQMRK AQS--QCWWE APVEELTLPQ IEQLKVSLEG LKMNVTKQAQ KLL
```
